# Suggestions to Improve the Comprehensibility of Current Definitions of Scientific Authorship for International Authors

**DOI:** 10.1007/s11948-019-00106-2

**Published:** 2019-04-23

**Authors:** Mohammad Hosseini, Luca Consoli, H. A. E. Zwart, Mariette A. van den Hoven

**Affiliations:** 1 School of Theology, Philosophy and Music, Dublin City University, All Hallows College, Senior House, Dublin, Ireland; 2 Faculty of Science, Institute for Science in Society, Radboud University Nijmegen, P.O. Box 9010, 6500 GL Nijmegen, The Netherlands; 3 Erasmus School of Philosophy, Erasmus University Rotterdam, Rotterdam, The Netherlands; 4 Department of Philosophy and Religious Studies - Ethiek Instituut, Utrecht University, Janskerkhof 13, 3512 BL Utrecht, The Netherlands

**Keywords:** Ethical authorship, Scientific authorship, Code of conduct, Metaphor, Virtue ethics

## Abstract

Much has been said about the need for improving the current definitions of scientific authorship, but an aspect that is often overlooked is how to formulate and communicate these definitions to ensure that they are comprehensible and useful for researchers, notably researchers active in international research consortia. In light of a rapid increase in international collaborations within natural sciences, this article uses authorship of this branch of sciences as an example and provides suggestions to improve the comprehensibility of the definitions of authorship in natural sciences. It assesses whether the definition of authorship provided by the European Code of Conduct for Research Integrity can deal with current issues and problems of scientific authorship. Notably, problems that are experienced in project groups with researchers coming from multiple countries. Using theories developed by Jürgen Habermas and Robert Merton, a normative framework is developed to articulate ethical authorship in natural sciences. Accordingly, enriching the current definition of authorship with normative elements and using discipline-specific metaphors to communicate them are introduced as possible ways of improving the comprehensibility of the definition of authorship in international environments. Finally, this article provides a proposal to be considered in the future revisions of the European Code of Conduct for Research Integrity.

## Introduction

Scientific authorship, as “the traditional vehicle of scientific communication”, faces an increase in the number and range of actors which it links (Gibbons et al. 1994, p. 34). This has resulted in an increase in the average number of authors per paper, the diversity of expertise involved in the writing of a paper and the fields and the types of organizations from which the authors originate. Consequently, the geographical distribution of these authors is also broadened (Gibbons et al. 1994). While recognizing these trends, this paper will focus on the growth of collaborations between co-authors coming from various countries (hereafter called international co-authors) and the challenges involved in the communication of the norms of authorship in these collaborations.

A key concern in communicating the norms of authorship is the promotion of *a definition of authorship* in academic environments. Often times, this definition is mentioned in institutional or international Codes Of Conduct (COCs) that provide a general description of what determines who an author is. These codes are the official tools in laying out professional responsibilities and explaining the limits of acceptability in academia (Davis 1999). In this article, the communication of results according to the prescriptions of COC is called good authorship. Ethical authorship, however, as will be described thoroughly, is beyond following prescriptions and concerns authors’ motivations for publishing.

As international co-authorships grow, defining scientific authorship in COCs becomes a complicated matter (LaFollette 1992; Gibbons et al. 1994; Cronin et al. 2003; Wagner 2006; Anderson et al. 2011; Anand 2015). For instance, historical and regional differences in the meaning and implications of the practice of authorship (Long 2001), language differences, cultural misunderstandings, management issues, differences in assumptions, expectations, roles and work styles indicate that in many instances, international co-authors are not always on the same page (Anderson 2010). Furthermore, due to differences in constituents of authorship in different countries and disciplines (Vasconcelos et al. 2014), variations in organization and management of research practices, research activities across academic, business and government sectors (Anderson 2010), or regulatory and legal differences (Bohnhorst et al. 2010), what is acceptable in one country might be considered problematic or even regarded as misconduct in another country. For example, in some countries, senior researchers may take credit for work done by junior colleagues, or in other cases, researchers may use authorship as a *quid pro quo*. In circumstances where some collaborators are more in need of publications, authorship credit might be distributed generously (or overly generously) to them with the expectation to receive similar favors in the future (Anderson et al. 2011).

Careful consideration of these issues is warranted by the rise of international co-authorships in recent years. In terms of the areas where this growth is more pronounced, data published by the U.S. National Science Board in 2018 are illuminating. Between 2006 and 2016, internationally co-authored publications grew from 16.7 to 21.7% of all co-authored publications worldwide. In particular, the natural sciences, notably astronomy with 54%, followed by geosciences, mathematics, biological sciences, mathematics, physics, and chemistry (all above the average of 24.2%), have the highest rates of international collaborations (National Science Board 2018). What is remarkable about this trend is that the growth in international co-authorships within these disciplines is independent of the presence of different authorship norms such as authors order, contribution types (Smith and Master 2017) and the average number of authors per publication (Abramo and D’Angelo 2015).

In light of the increasing incidence of international co-authorships in the natural sciences and the difficulties involved in defining and communicating good authorship, this paper aims to enrich definitions of authorship, so that they are less likely to cause misinterpretation among international co-authors in the natural sciences. It is worth mentioning that by focusing on the natural sciences, this article is neither indicating importance nor primacy of this branch of science over others. This is a pragmatic choice given that in this branch of sciences, international collaborations are growing more rapidly compared to other areas.

This paper is structured in the following way: it will begin by detailing some practical difficulties of communicating a clear definition of authorship to international co-authors via COCs. Using an internationally accepted code of conduct, it assesses how the provided definition responds to challenges faced by international groups. With this in mind, the normative foundations of the concept of authorship in the natural sciences will be identified. This will help to develop a theoretical framework to explain what ethical authorship in the natural sciences entails. The third part focuses on the communication of the suggested framework and its normative elements and provides an example that can be used to explain ethical authorship in international environments. Finally, this article concludes that the suggested framework combined with its effective communication might enhance the usefulness of the definition of authorship and make it less vulnerable to misinterpretation.

### Challenges Involved in the Communication of the Norms of Good Authorship via COCs

As mentioned in the introduction, academic COCs often entail a definition that explains the boundaries of good authorship and requires the compliance of scientists. Besides complexities involved in the formulation of a definition of authorship which is unequivocal for scientists worldwide, the normative authority and usefulness of COCs are often questioned as well. For instance, it is argued that one fundamental issue with communicating norms via COCs is that the scientists often see COCs as an “external, superimposed, artificial and unrealistic” document that provides formalistic frameworks, with poor relevance to daily practices (Consoli 2008, p. 240).

On the one hand, definitions of scientific authorship are complicated to articulate (Biagioli and Galison 2003), as what constitutes authorship differ per period and discourse (Foucault 1979), and, given the growth of international co-authorships, in practice, they imply different meanings for authors from other countries. On the other hand, instruments which do attempt to communicate such definitions (i.e. COCs) are often seen as containing too many abstract and formal elements to be practically useful, and too general for and external to daily practices (Forsberg et al. 2018).

As such, to explain the difficulties of formulation and communication of a definition of authorship that will be comprehensible to international co-authors, analyzing one of the current definitions of authorship will be helpful here. For that purpose, due to its international character and broad application, the European Code of Conduct for Research Integrity (ECCRI) is chosen. The publisher, All European Academies (ALLEA) consists of 59 Science Academies based in more than 40 countries (ALLEA 2017), and thus, this code epitomizes an internationally recognizable guideline that is drafted with the support and cooperation of academic institutions from several countries.

In its latest version published in March 2017, ECCRI mentions *publication and dissemination* as an important aspect of good research practice. Issues such as sequence of authorship, timely, open, transparent and accurate communication to the general public, acknowledgment of the important contribution of others and disclosure of conflict of interests are also mentioned. More importantly, ECCRI encourages authors to acknowledge that authorship is based on “a significant contribution to the design of the research, relevant data collection, or the analysis or interpretation of the results” (ALLEA 2017, p. 7).

In this definition, significant contribution to named activities seems to be the *necessary and sufficient* prerequisite for/of becoming an author. Given the aforementioned growth of international collaborations, however, different interpretations of *significant contribution* are likely to exacerbate current ambiguities and cause more tensions. The term significant has dissimilar meanings for different contributors, and given the participation of international co-authors with various working cultures and habits, it will contribute to the misunderstanding of good authorship. For example, providing feedback in a short meeting that leads to a major change of direction in the project could be seen as a significant contribution to the project and deserving authorship status by some. This way of receiving authorship status might feel unwarranted by other contributors who spent months conducting experiments or analyzing data.

Clearly, granting authorship status to those who have not contributed *significantly* to the work conveys benefit to them, but at the same time, it also reduces the appropriate benefit to those who actually contributed to the work and impedes a fair distribution of credit (Strange 2008). Therefore, a misunderstanding of the notion of significant contribution is likely to contribute to authorship abuse (Strange 2008), and this suggests that the notion of significant contribution alone seems to be ill-equipped in addressing disagreements about authorship in international projects.

That said, it is also safe to assume that, due to the discrepancies in what constitutes authorship in different disciplines (Larivière et al. 2016), creating imperatives that are both accurate and general enough to cover all the possible violations of the norms of authorship for all disciplines in the natural sciences would be impractical. Accurate descriptions that encompass every violation in every discipline would make the definition not only longer but even more perplexing and far more difficult to comprehend for international co-authors. In other words, while the current definition is open-ended and subject to abuse, strengthening it by adding all the necessary descriptive elements is neither feasible nor non-problematic.

**Fig. 1:**
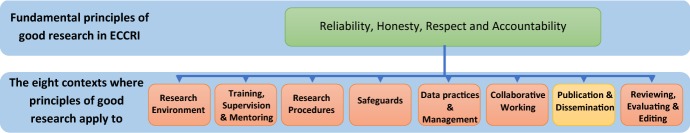
European code of conduct for research integrity, fundamental principles of good research and the contexts where those principles apply to

The developers of ECCRI too seem to be aware of these issues and have subsumed publication and dissemination as one condition of good research practices, which together with seven other contexts are based on the fundamental principles of research integrity. These principles include reliability, honesty, respect, and accountability (see Fig. 1). ECCRI asserts that these four principles “guide researchers in their work as well as in their engagement with the practical, ethical and intellectual challenges inherent in research” (ALLEA 2017, p. 4).

This article argues that, given the specific and complex issues pertinent to authorship, some of these fundamental principles seem to be detached from what a robust definition of authorship requires. For example, the way in which the principle of respect is currently qualified in ECCRI [“respect for colleagues, research participants, society, ecosystems, cultural heritage and the environment” (ALLEA 2017, p. 4)] is likely to cause confusion among international co-authors. In particular, in some cultural contexts where “it may be deemed appropriate or even required to give authorship credit out of respect (e.g. to senior colleagues, or directors of institutes)” (Smith et al. 2014, p. 5), the notion of respect for colleagues is prone to cause serious problems in the context of authorship. Similarly, the principle of honesty, that entails “honesty in developing, undertaking, reviewing, reporting and communicating research in a transparent, fair, full and unbiased way” (ALLEA 2017, p. 4), is likely to be affected by organizational policies and their values of openness and release policies. The Human Genome Project is a particularly good example of cooperation between scientists that, due to their affiliations, revealed “philosophical differences concerning data access and release policies” (Resnick 2007, p. 16). In that project, while the international consortium was committed to granting free access to information, Celera, a private firm that held the patent to the *shotgun approach*,^1^ was against the free and rapid release of DNA sequence data, to ensure that they could charge users a fee for results (Resnick 2007).

**Fig. 2:**
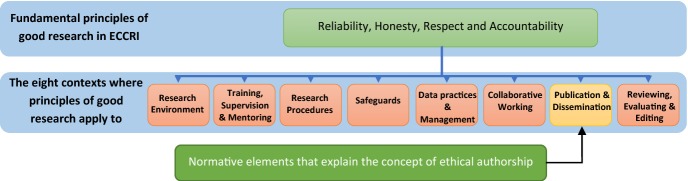
Given its complexities, authorship requires different ethical principles and normative elements that should be directly linked to the context of publication and dissemination

As analyzing ECCRI and scrutinizing its definition of authorship has shown, scientists who argue that these definitions are too formal and abstract seem to have a point, because they are difficult to apply in daily practices. One alternative solution to address this issue is to combine the current definition of authorship with specific normative elements that further elaborate on the notion of ethical authorship. In other words, instead of only basing the definition of authorship on *who is/deserves to be an author (i.e. the one who has made a significant contribution)*, and remotely linking that definition to the fundamental principles of research integrity that are not always relevant to the realities of scientific authorship, this article suggests adding separate normative elements to this definition, which are relevant to the concept of ethical authorship. Doing this not only explains *who is an author*, but also readily clarifies *who is an ethical author* (see Fig. 2).

Using this method seems to take the stress away from defining authorship with *the extent of contribution* as the major constituent of the author function (that differs per period and discourse, and is particularly difficult to define in international contexts), to providing a more comprehensive picture that also entails the characteristics of an ethical author (depending on what authorship may mean in the given disciplines and time). Providing a definition that is enriched with these normative components will potentially also give guidance in relation to conflicting interests/perspectives (e.g. on who deserves to be a co-author) on authorship in practice through using underlying norms of practices of authorship.

## Enriching the Definition of Authorship with Normative Concepts

In light of challenges faced in providing a definition of authorship that copes with the complexities of modern science, this article suggests using normative elements in addition to (more) descriptive elements in defining authorship. As mentioned in the introduction, while defining authorship is complex, the presence of international co-authors who come from various institutions and cultural backgrounds make the task of explaining what authorship entails even more difficult. Therefore, through delineating the characteristics of a*n ethical author*, a new method will be suggested to articulate the concept of authorship in ways that will make the provided definition in COCs more accessible and useful in practice. To that end, this section will first propose a theoretical framework to extract the normative elements involved in determining what ethical authorship is in the natural sciences. Later, means of communicating those norms will be suggested. These suggestions aim to decrease the likelihood of making dissimilar interpretations from the definition of authorship, and make them more practical and relevant to international collaborations.

### Normative Elements in Authorship in the Natural Sciences

Scientists working today have a myriad of options for the publication of their work. Since the scientific community is best placed to judge the merits of authored science and to provide critical remarks (Burns et al. 2003), identifying relevant norms and moral ideals of authorship in international peer-reviewed journals will be the focus of this article. To that end, discussions about the process by which nature is observed and claims about it communicated should be considered. To understand this process of observation and communication, this article will use theories developed by the German philosopher and sociologist, Jürgen Habermas. As will be shown shortly, his theories help unpack the social context in order to explain and assess the behavior of involved scientists by making references to human interests and the conditions of objective knowledge.

What makes Habermasian theories particularly suitable for the identification of the conditions of being an ethical author is the fundamental normative approach he takes regarding knowledge, asserting that since knowledge is produced by subjects, its status depends on subjective conditions. He rejects the notion that the only norm for evaluating and criticizing scientific knowledge is the factual development of science (Habermas 1971). In terms of the natural sciences in particular, he notes that while the conditions guaranteeing the objectivity of human experience (i.e. technical correctness) help explore reality by objectifying it, discursive verification (i.e. being verified in a discourse and corroborated in argument) is the necessary condition for reaching consensus on the claims to truth, which “reflects the intersubjective validity, on the basis of which something may be predicated of objects of experience” (Habermas 1973, p. 167).

Additionally, Habermas’ views on the ideal (reasonable, transparent, egalitarian) community seem especially relevant to the issue of authorship in science. Building on the work of C.S. Peirce, he considered the scientific community as an almost ideal speech community, as an approximation of the normative ideal of communication where (via anonymous peer review and other techniques) manipulation and strategic communication is abolished, hierarchies do not count and no other force is at work than the force of the stronger argument (Zwart 2001).

Given the role of scientific authors in communication of claims about nature, using Habermasian views seems to be crucial to the current context as these can help lay out what it means to be an ethical author based on an author’s role in producing objective scientific knowledge. In doing that, this article uses a dichotomy that was suggested by Habermas in the 1970s and will develop it further using his later theories on the communicative act.

By linking knowledge to subjects and the qualities and conditions of the system of social labor, Habermas makes a distinction between *reflective* and *productive* knowledge. He asserts that productive knowledge is the type that does not question its own epistemological foundation and hence, lacks self-reflection. Reflective knowledge, on the other hand, is the result of genuine discourse and reflection (Habermas 1971). Thus, one necessary condition of producing objective scientific knowledge is that it should *not* be merely produced for the sake of having produced knowledge. Those who produce and communicate it ought to be interested in having their methods questioned and aim to have their results challenged and validated by other scientists. Putting that in the context of scientific authorship implies that being published is not the ultimate aim of objective scientific knowledge and producing output should only be a means for inviting further reflection.

Furthermore, as mentioned at the beginning of this section, Habermas believes that the objectivity of human perceptions or sensory experiences of nature “consists precisely in its being intersubjectively shared” (Habermas 1973, p. 168). As such, the other necessary condition for the objectivity of scientific claims (that separates them from *happenings* or *state of affairs*) is to achieve consensus on them (Habermas 1973). This suggests that a prerequisite of producing objective science is that it should be shared and communicated within the scientific community, to verify claims through sufficient questioning and specification. In his later work, Habermas qualifies the conditions of this communication via the notion of genuine discourse.

In explaining the conditions of good communication, Habermas provides a distinction that clarifies what genuine discourse means. He does this through distinguishing communicative from purposive-rational (strategic) actions. According to him, understanding necessitates communication, but motivations for communication can either be *communicative*, aimed at understanding, or *strategic*, aimed at gaining power and domination (Habermas 1996). Thus, in order to be communicative rather than strategic, in addition to its technical correctness, objective scientific knowledge should be communicated with the aim of becoming self-reflective and should involve genuine discourse aimed at understanding without having other strategic aims.

On the basis of this understanding of scientific knowledge, normative elements of authorship that contribute to the production of this type of knowledge can be extracted. One way of doing this is to start with the functions of authorship for science and highlighting the required normative features that result in the production of objective scientific knowledge.

### Using the Function of Authorship to Move from Objective Science to Ethical Authorship

Given the emphasis that Habermas puts on conditions of communication for the production of objective scientific knowledge, his theories can guide this article in delineating the normative aspects involved in authorship in the natural sciences. To practice authorship in accordance with Habermasian ideals implies communicating perceptions of natural phenomena to other scientists *only* with the purpose of validating conclusions and aimed at improving understanding. That is to say, from the perspective of authoring scientists, the aims of communicating results ought to be *becoming self*-*reflective*, and improving the scientific community’s *understanding* of nature via genuine discourse.

Yet, these ideals put forward by Habermas expose scientific authorship to a new tension. While important normative elements of ethical authorship can be extracted from Habermasian theories, they seem rather general (be communicative rather than strategic) and may not be immediately practical or realistic. Moreover, highlighting self-reflection and initiation of genuine discourse for understating nature, as legitimate conditions of producing objective science, seems to satisfy the interests of one of the stakeholders of scientific endeavor (namely *science*), and the interests of two other important stakeholders, namely *scientists* and *scientific institutions*, seem to remain unaddressed.

From a scientist’s point of view, authorship may have several other functions. As argued by sociologists of science, scientists make knowledge available to others in exchange for credit (Latour and Woolgar 1986; Bourdieu and Nice 2004). In academic environments, personal reputation, professional success, and, consequently remuneration, are tightly coupled with publication salience and citation (Cronin 1996). Being an author yields recognition, and scientists who publish more frequently are more likely to succeed in academia (Resnik 2010). Thus, in a very real sense, the professional identity of scientists is in large part constituted through the way in which they perform as an author. If that is true, the awarded credit and its importance for academic life would prevent scientists from being focused only on becoming self-reflective or improving understanding through genuine discourse.

Furthermore, in relation to institutional interests, the quantity of publications and citations analyses play a crucial role in the evaluation of institutional activities, allocation of funds (Bornmann et al. 2008), and also universities’ position in modern ranking systems (van Raan 2005). Therefore, academic institutions need scientists that can publish and attract funding for future research (Resnick 2007). This means that having self-reflective scientists who are only involved in discussions leading to improving understanding about nature is not necessarily conducive to institutional interests.

While career incentives and aspirations for climbing the academic ladder are among “forms of positive reinforcement” for scientists (Resnik 2010, p. 68), a one-sided focus on the eagerness of scientists to receive credits for their contribution to the production of objective science in terms of quantifiable performance indicators is problematic (Forsberg et al. 2018). Similarly, given the links between allocated funds and quantity of publications, reproaching scientists and scientific institutions for having strategic interests seems to ignore the fact that in actual practice, the scientific ecosystem is confronted with an inextricable mix of interests. Evidently, scientific institutions encourage publication so that they can continue to exist and compete with other institutions. A categorical enforcement of norms of good science as put by Habermas seems too far removed from actual practice. Rather than strengthening integrity robustness, it could demotivate scientists and eventually jeopardize the openness of science and hamper allocation of funds to scientific institutions.

Given that Habermas too is not an absolutist and only criticizes the *exclusive application* of strategic reasoning at the expense of communicative reason (Radder 2012), it is safe to assume that in this context, and given the mentioned considerations in terms of scientists’ legitimate interests in publishing and receiving credit, a *reasonable* extent of productivity would not be inconsistent with Habermasian ideals. Quantifiable output measures may be employed as performance indicators, as long as the awareness prevails that the ultimate goal of publishing is genuine communication, in other words as long as the performance indicator does not become a goal in itself at the expense of reflection and self-reflection.

**Fig. 3:**
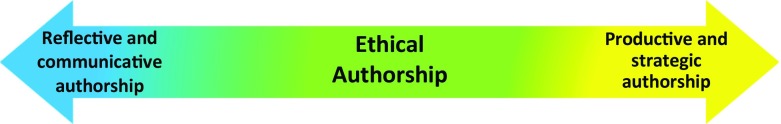
State of reasonableness in the communication of scientific knowledge

From scientists’ and scientific institutions’ points of view, a reasonable extension of productivity is a state where the right balance between reflectivity and productivity regarding their reasons for publication, and also between communicative and strategic motivations for communication of their results exists. One way to find this balanced state is to place the conditions of objective scientific knowledge on a spectrum with productive/strategic and reflective/communicative features at the two ends, and introduce the middle of the spectrum as the desired point to aspire in the real world. This middle point can be called the state of *reasonableness in productivity and communication of scientific knowledge* (see Fig. 3).

One could also formulate this in Aristotelian terms, arguing that ethical (virtuous) authorship is the mean between deficiency (i.e. insufficient emphasis on the importance of sharing knowledge through scholarly publications) and excess (i.e. publishing merely to boost performance indicators).

Given the critiques that were leveled against the ECCRI’s definition of authorship in this paper, identifying a new tension about the state of *reasonableness in productivity and communication* is not very difficult. One can correctly argue that similar to the notion of ‘significant’ in the context of ‘significant contribution’, ‘reasonable’ in the context of ‘reasonableness in productivity and communication’ is open-ended and requires further qualification to prevent misunderstanding in international co-authorships. Thus, although Habermas helps this article in identifying the basis and contours of ethical authorship in the natural sciences, using his theories contributes to a new practical tension. Namely, yet another open-ended statement (i.e. reasonableness in productivity and communication) is being used to explain what is it to be an ethical author. In terms of (Aristotelian) virtue ethics: the difference between reasonable and unreasonable, between sufficient, insufficient and excessive, cannot be defined exactly (in quantitative terms) but is rather a matter of attitude and ethos, which is missing here.

One way to address this problem is to turn to other sources that, while sharing a core fundamental basis with Habermasian theories, are more pragmatic in dealing with issues that are linked with personal and institutional gains from publications. Given the tight links of authorship with the reward system of science (Biagioli and Galison 2003), using theories that acknowledge the dependence of science on its reward system seem to be helpful in articulating what is meant by *reasonableness in productivity and communication of scientific knowledge*.

To be more precise, one way to clarify the notion of reasonableness in this context is to admit the dependence of science on its reward system without considering it entirely problematic. As explained earlier, since the gained credit encourages scientists to discover nature and communicate their results, expecting authors to solely publish for the sake of improving science without thinking about their personal interests is not realistic. To that end using the notion of ‘ethos of science’ developed by the American sociologist Robert King Merton will be beneficial. The ethos of science entails values that can help in providing a definition of an ethical author by painting the picture of a scientist who is “detached, methodical and committed to the search for the truth rather than personal glorification” (Macfarlane and Cheng 2008, p. 69). These values will be explained in more detail below.

### Mertonian Values, Corrective to Habermasian Ideals and Familiar for Scientists

In what follows, a short introduction on Mertonian ethos will be followed by more specific justifications for their usefulness in the current context. As will be discussed in detail, the ethos of science fine-tunes Habermasian ideals and improves the normative framework that was developed in the previous section.

In 1943, Robert Merton presented four values of scientific research, including Communism, Universalism, Disinterestedness and Organized Skepticism, also known through the acronym CUDOS.

*Communism* Merton considers science as a cultural heritage that belongs to the community and public domain and invites scientists to acknowledge the effect that formerly discovered knowledge has had on their current progress (Merton 1973).

*Universalism* Deeply rooted in the impersonal character of science, universalism in science suggests that acceptance or rejection of scientific claims should never be dependent on the race, nationality, religion, class or other social and personal qualities of the author and all such qualities are irrelevant (Merton 1973).

*Disinterestedness* As a basic institutional element, the demand for disinterestedness has a firm basis in the public and testable character of science and contributes to the integrity of researchers. It epitomizes respect for peers, science and society, and manifests scientists’ accountability (Merton 1973).

*Organized skepticism* Interrelated with all the three aforementioned values, skepticism and scrutiny of beliefs are considered as a methodological as well as an institutional mandate. Merton postulates a relationship between organized skepticism and the other three ethos, and implicitly assigns a supervisory role to it (Merton 1973).

In addition to their advantages for describing the desired values of scientific research, for the following reasons, Mertonian ‘ethos of science’ are beneficial within the context and corrective to the normative spectrum that was created using Habermasian ideals:

1. Complying with the fundamental assumptions made by Habermas: Merton asserts that unlike technical methodologies that are a matter of expediency due to rational reasons, the ethos of science is binding because it is believed to be right and good (Merton 1973). This clearly overlaps with the Habermasian fundamental assumption that scientists need to follow certain norms (e.g. willingness to communicate) because otherwise subjective or strategic features may contaminate the knowledge produced.
2. Corrective to Habermasian ideals: Merton recognizes the reward system as a necessary feature of the conduct of science and believes that it prevents science from sinking into anonymity, and “reinforces and perpetuates the institutional emphasis upon originality” (Merton 1973, p. 302). Thus, using ethos of science responds well to the shortcomings of the Habermasian normative framework by admitting that science, scientists and scientific institutions are dependent on a reward system, and hence it is not problematic per se that prolific productivity tends to be beneficial in science.
3. Relevant to the current practice of science: In practice, if correctly understood, Mertonian values “remain at the heart of the production of reliable knowledge” (Cottey 2016, p. 369). Despite being originally suggested decades ago, Mertonian values are still highly popular among modern scientists. In fact, scientists in different disciplines and levels of seniority feel *to a great exten*t that those values “*should* represent the behavior of scientists” (Anderson et al. 2010, p. 385).
4. Useful for international contexts: Particularly useful for the current context and communication of the norms of ethical authorship in the natural sciences to international co-authors, is that like other general concepts, Mertonian values possess a nonlocal meaning, and hence, they can be applied in various new situations (Radder 2010). Given the fluidity of the concept of authorship, notably, because its meaning differs per period and discourse (Foucault 1979; Zwart 2001, 2005), this nonlocality of Mertonian values will strengthen the suggested concept in dealing with the disciplinary contingencies and international norms in a meaningful manner.

**Fig. 4:**
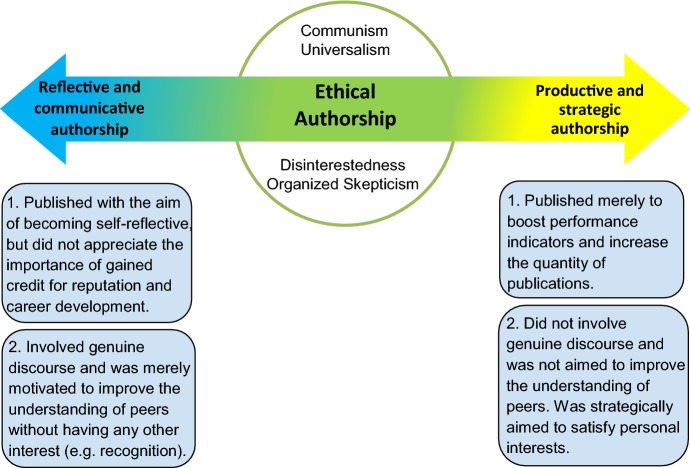
The ethos of reasonableness in the communication of scientific knowledge

Thus, Mertonian values are well equipped to fine-tune the normative elements that were extracted from Habermasian theories. To create the hybrid between Merton and Habermas (the so-called ethos of reasonableness in authorship) the boundaries of ‘reasonableness in productivity and communication’ will be limited with Mertonian values. That yields a normative continuum with a green zone in the middle that represents reasonableness in productivity and communication (the “mean” of virtue ethics). This article argues that this green area entails the essence of what it is to be an ethical author in the natural sciences (Fig. 4).

Now that some normative elements suitable for the definition of authorship in the natural sciences are identified, the next challenge is to suggest methods for the communication of these to international co-authors. As mentioned earlier, one way of communicating these definitions at the moment is via COCs such as the ECCRI, but given the challenges of communicating norms of authorship to international co-authors, a new approach in formulating and communicating these normative elements will be suggested.

## Communicating the Ethos of Reasonableness in the Authorship of Natural Sciences

In the last section, a hybrid solution was used that was a combination of theories developed by Habermas and Merton. It was concluded that in the natural sciences, scientists must publish the results of their observations, experiments, and analyses with the aim of becoming self-reflective and improving the understanding of the scientific community, without exclusively thinking about their own interests, through adhering to the ethos of science as put forward by Merton.

The next challenge is to find effective methods for communicating these abstract normative elements to international co-authors in a way that is sensitive to differences in terms of research cultures and conventions between disciplines and global regions. Given the challenges of providing descriptive prescriptions that are useful in daily practice, this article suggests the use of relevant metaphors as an effective way of explaining the ethos of reasonableness in the communication of scientific knowledge. To further understand the role that metaphors can play in the communication of norms of authorship, the following two sections will explain the advantages and challenges of using metaphors in the current context.

### The Power of Metaphor

This section will stress the usefulness of metaphors in explaining the norms of ethical authorship of the natural sciences to international co-authors, and suggests that using metaphors in parallel with descriptive prescriptions of the COCs is likely to improve the understanding of international co-authors about these norms.

It is argued that metaphors can be used as interpretive conceptual resources that allow one to understand the logic and entailments of concepts (Johnson 2008). Hence, using them in addition to the current definition of authorship could be helpful in the prevention of confusion and misunderstanding among international co-authors. The importance of “metaphor as a methodological resource is in its capacity to render and connect knowledge” with the lived experiences (Black 2013, p. 26). The imagination involved in using metaphors reaches deep into “the hard stuff of the world of physical and social experience and seizes upon possible new relations for thought and action” (Fesmire 2003, p. 66).

Furthermore, since the perceptions and inferences that follow from the use of metaphors are believed to benefit actors in both understanding and making the appropriate action (Lakoff and Johnson 2003), using them in COCs does not seem inconsistent with the envisaged role of these documents. In fact, several empirical studies have highlighted the positive effects of using metaphors. For instance, 15 years after Mark Johnson’s claims regarding the historical uses of metaphor in improving human moral understanding and advantages in explaining ethical concepts (Johnson 1993), empirical research in brain science and neural computation attested to positive influences of metaphor on basic operation of abstract thinking (Johnson 2008) and processing of abstract concepts (Lakoff 2008). More importantly, the possibility of creating universal metaphors that do not use cultural information and hence, are not culture-specific (Lakoff and Johnson 2003) is arguably the most unique advantage of using metaphors to communicate the norms of ethical authorship to international co-authors.

This article recommends that the notion of ethos of reasonableness in the communication of scientific knowledge provides the necessary conceptual substance to what needs to be defined as ethical authorship, and with the help of metaphors, this concept becomes more accessible to international co-authors. That said, due to the disciplinary differences in what constitutes a reasonable degree of productivity and a balance between reflective and productive reasoning for publications, field-experts and supervisors are best placed to develop and/or revise relevant metaphors based on what ethical authorship means in their discipline. This could be based on the disciplinary differences in relation to units of publications. Best articulated by the English physicist and historian of science Derek J. de Solla Price, expected productivity varies in different disciplines. For instance, while some branches of biochemistry can produce a separable and valid publication in a few weeks, some parts of astrophysics require 2 or 3 years of work before they can publish a valid unit (Price 1981). Thus, it is reasonable to argue that only field-experts and supervisors who are interested in promoting ethical authorship, could help in articulation and translation of the state of reasonableness in the communication of scientific knowledge within their disciplines.

### Challenges of Using Metaphors

It is worth mentioning that despite the aforementioned considerations in the usefulness of metaphors, using them in explaining moral issues is also criticized. Two of the more relevant critiques in this context will be mentioned here. Firstly, due to their poetic and rhetorical nature, they are believed to have no rational or conceptual content and merely represent an “emotional expression of approval or disapproval for certain acts or states of character” (Johnson 1993, p. 34). Secondly, metaphors are believed to be cognitively indeterminate, contributing to ambiguity and multivalence, hence, making it harder to find the right action for a given situation (Johnson 1993). In what follows, measures that were taken in this article to safeguard its recommendation against both lines of critique are explained.

In response to the first critique, since the main suggestion of this article is based on a reasonably solid normative framework, and uses metaphors to further explain the extracted normative concept (i.e. ethos of reasonableness in the authorship of natural sciences), developed metaphors will not be irrational or non-conceptual. Regarding the second critique, given that this article suggests using metaphors that are developed by field-experts and in addition to descriptive definitions, metaphors aimed at explaining the suggested concept of ethical authorship are likely to trigger similar moral values for co-authors independent of their nationality or culture. Thus, with the use of metaphors that allow scientists to infer similar normative values, prescriptions are less likely to be misinterpreted in a multinational environment.

The next section will introduce a metaphor that can better explain the notion of ethical authorship in the natural sciences based on the normative framework that was suggested in this article.

### An Example of a Metaphor to Explain Ethical Authorship in Natural Sciences

This example is inspired by the way in which Habermas explains the role of communication in understanding nature. In describing the normative aspects of the communication of claims in natural sciences, Habermas notes that through communicated claims, readers understand a symbolic pre-structured representation of reality, and attempt to take the same position the scientist(s) adopted in describing their perception of nature. Once the observer shares an observation, the interpreter experiences a communication: a symbolically established relationship with the observer. While both observer and interpreter are related to sectors of reality, the first has an *immediate* experience and the latter a *mediated* experience (Habermas 1971).

The idea that the observer has an immediate access to observable events while interpreters have a mediated access suggests that in empirical-analytic natural sciences where scientists express the details of one of their analyses/experiments about the natural world, communication of claims with other scientists can be seen as a metaphoric *testimony in a court about a witnessed event*. In this metaphor, the communication addresses an event that happened in the natural world, and the scientist(s) who observed the natural event are metaphorically acting like a *voluntary witness*.^2^ Consequently, the scientific community will act like a jury that will also listen to other witnesses, acquire relevant expertise (from reviewers), and make a decision about these claims (see Table 1). This article argues that in communicating the natural sciences, an ethical author should act like a voluntary witness. Even though the use of witnesses in judicial systems varies worldwide, which might affect how this metaphor is understood, a slightly fine-tuned version of this metaphor might work in an international community, because it primarily emphasizes the process of witnessing and testifying. Using the metaphoric voluntary witness who has observed an event and testifies about its details in a court is a useful way of explaining the ethos of reasonableness in the communication of scientific knowledge.

**Table 1:** Stakeholders in scientific authorship and their role in the suggested metaphor

Scientific authorship in the natural sciences	Voluntary witness as a metaphor
Authoring scientist(s)	Witness(es)
Experiments/analyses	An observable event
Doing the experiments/analyzing	Witnessing
Scientific community	A jury of peers
Authorship in the natural sciences	Testifying

In relation to complying with the suggested framework, the voluntary witness satisfies the requirements of ethos of reasonableness:

As this article argued, from the perspective of ethical authors, the aims of authorship of results in the natural sciences ought to be (a) becoming self-reflective, and (b) improving the scientific community’s understanding of nature via a genuine discourse. Therefore, a good voluntary witness in this metaphor needs to fulfill both of those conditions too. Since a voluntary witness is under no obligation to testify, it is safe to assume that she is genuinely interested in communicating observations. Furthermore, given that in most cases there are also other witnesses (whether voluntary or not) who could have seen the same event from a different perspective, or with dissimilar means (e.g. color vision deficiency affects how one sees colors), the mere presence of a voluntary witness in the hearing suggests that one is there to have her observations confirmed and recognized. Through having one’s testimony compared with testimonies provided by other witnesses, one will contribute to a genuine discourse, and finally, once judged by a competent jury, one will become aware of the flaws/misinterpretations in one’s own testimony.

Also, in relation to the Mertonian ethos of science, the metaphoric voluntary witness remains relevant and meets the requirements of the normative framework. Regarding universalism, a good witness’ claims are not dependent on personal criteria such as race, nationality or religion. One can be a witness as long as the person involved is capable of knowing, and shows that she has indeed carefully observed the event about which she testifies. Communism would imply that the testimony belongs to the public domain and the jury, lawyers, prosecutors, or other witnesses are free to use the testimony that was provided by the voluntary witness as long as they mention the source. The focus is on sharing and transparency, rather than on secrecy. A good voluntary witness is also likely to be disinterested in what the ruling will be, and although interested in the topic and eager to have her observations and points of view recognized, ideally, she would not favor a side in the court. Organized skepticism would imply having a skeptical attitude towards what is being discussed. Good voluntary witnesses would not accept what they hear on its face value and will be critical about the provided facts and arguments by other witnesses.

In explaining the concept of ethical authorship, using the notion of a *good voluntary witness* and asking scientists to consider this as a model addresses several challenges involved in international collaborations. Perhaps highlighting a crucial similarity between authorship and witnessing explains this more clearly. Although at first glance authorship may seem a clear concept, on further inspection multiple ambiguities arise, as we have seen, due for instance to cultural and conventional differences of authorship between cultures or research fields (e.g. different answers to questions such as: who should count as an author, what are the responsibilities of an author, etc.). The witness concept is similar to authorship in this sense. Although it may initially seem straightforward, the precise meaning of the concept, as well as the obligations involved, may differ depending on the context. For instance, because witnesses play different roles in inquisitorial compared to adversarial systems, they might have dissimilar obligations. The term ‘witness’ may have a specific meaning (witnessing a concrete event) but also a more general meaning (we are all ‘witnesses’ of what is happening in the world today), and this can be discerned in authorship as well, for authors can be authors of specific publications, but also authors in a more general sense (professionals who make a living from academic writing and contribute to academic discourse). Still, this article argues that in being a witness (as well as in being an author) a number of quintessential values are involved, such as, for instance, trustworthiness and impartiality, even if the precise role and function of witnesses (or authors) may differ in various contexts and traditions. That said, because the witness is a familiar concept, it is more likely to trigger similar moral values. Thus, it is safe to assume that by asking the question ‘how does a good voluntary witness act?’, ‘with honesty’, ‘objectively’ or ‘neutrally’ will be among the first mentioned qualities. Furthermore, due to its legal implications and use, a witness is likely to come across as an important function, which comes with certain responsibilities, one of which entails ‘respect’ for the subject of inquiry and those involved. A witness is valued for her observations as a ‘reliable’ participant and can be held ‘accountable’ if necessary.

In terms of the limitations of this metaphor, depending on the context one can think of factors that restrict its applicability. For instance, in countries where the public trust in the legal system is rather low, or countries where the adversarial legal system is used, the role of witness might be interpreted slightly differently, and hence, this metaphor might not be as useful as other places. Furthermore, a witness (no matter how well-intended or voluntary) might misremember facts, causing the testimony to become inaccurate, and an opportunistic person might try using this as an excuse for justifying dishonest reporting. It should also be clear that this metaphor is not going to benefit disciplines that do not require direct observations or empirical research.

## Conclusion

Given the rapid growth of international co-authorships, this article provides suggestions to improve the comprehensibility of current definitions of authorship for international co-authors. Via laying out the dynamics of modern authorship practices and analyzing the definition of authorship provided in the ECCRI, it was argued that this definition could be misinterpreted and misunderstood. Hence, enriching the current definition using normative elements that are specific to the practice of authorship was suggested. Due to the rapid growth of international co-authorships in the natural sciences, this branch of science was used as an example to create a framework for extracting the normative elements involved in the authorship of claims about nature.

Using theory of communication and human interests in science developed by Habermas and Mertonian ethos of science, it was argued that scientists would ideally use publication as a means for facilitating understanding and validation of their results. In doing that, furthering scientific careers and benefitting from the fruits of scientific publications through gained credit is not necessarily unethical as long as scientists adhere to the ethos of science. Finally, it was suggested that using metaphorical statements that were developed by field-experts and supervisors on the basis of the suggested framework, provides a platform for communication of norms of authorship and their importance within international project groups.

Without empirical research to determine the effectiveness of using metaphors, no one can claim with certainty that by using metaphors misunderstanding of norms will be completely prevented. What this article claims is that given the normative aspects that are involved in determining authorship in the natural sciences, using metaphors makes these norms less likely to be misinterpreted, and hence, in combination with current definitions, informs scientists about the ideals that are necessary for ethical authorship. In addition to improving the comprehensibility of open-ended definitions for international groups, using metaphors extends the reach of COCs, and provides space for conversation and self-reflection. A metaphor becomes the *memory*, the kind of educational takeaway message that scientists need to carry in order to deal with the moral question of future circumstances.

Therefore, in addition to the current definition of authorship provided by ECCRI:

All authors agree on the sequence of authorship, acknowledging that authorship itself is based on a significant contribution to the design of the research, relevant data collection, or the analysis or interpretation of the results (ALLEA 2017, p. 7);

this article suggests considering the inclusion of the concept of ethical authorship and describing the state of reasonableness in the communication of scientific knowledge and noting the usefulness of metaphors in explaining this state. One proposal to consider and use in the ECCRI could be:

Ethical Authorship refers to authors’ motivations for publications, the best of which entails using publications for validating methodology and results, and improving the understanding of the scientific community regarding the discussed issue. Using metaphors or other creative methods, supervisors and field-experts explain the concept of Ethical Authorship to their colleagues. One useful metaphor to explain what ethical authorship means in natural sciences, is to encourage authors to behave like a voluntary witness who witnessed an event in nature, and aims to testify about its details to the scientific community.

Conducting empirical research among co-authors of different nationality/discipline to determine the success rates of using different metaphors in improving the comprehensibility of authorship definitions could be among useful topics for future research.

## Footnotes

1 “Instead of tediously sequencing the genome one piece at a time, Venter had the idea to break apart the entire genome, sequence the pieces, and put the pieces back together using algorithms run on supercomputers to match the pieces” (Resnick 2007, p. 15).

2 A witness willing to testify in court without the need for a subpoena compelling the witness to appear. Accessed March 30, 2018 Retrieved from the law dictionary (https://dictionary.thelaw.com/voluntary-witness/).
